# Paths to cheminformatics: Q&A with Phyo Phyo Kyaw Zin

**DOI:** 10.1186/s13321-022-00668-7

**Published:** 2023-02-13

**Authors:** Phyo Phyo Kyaw Zin

**Affiliations:** Terray Therapeutics, Inc., Pasadena, CA 91106 USA

## Introduction by the Editors-in-Chief

Recently we described [1] an initiative to put a spotlight on diversity within the cheminformatics community. As part of that we initiated a series of interviews, and this article is the second in that series. Phyo Phyo Kyaw Zin, Ph.D (Chemistry), is a Cheminformatics Scientist at Terray Therapeutics, where she develops in-silico techniques to advance drug discovery. She is a 2019 AAUW international doctoral fellow. She has research expertise in Cheminformatics, particularly in developing tools and algorithms to enumerate and design large, diverse/focused in-silico chemical libraries for drug design and optimization, automated pipelines to efficiently mine, curate, and process chemical/biological data, SAR analysis, and building predictive machine learning models for chemical/biological endpoints including ADME/Toxicity.

## What has been your path to where you are today?

PPKZ: I came from humble roots in Myanmar. Since childhood, I had ambitions of becoming a scientist and inventor. I took a couple of years off after high school to do various volunteering work, including assisting the blind, teaching elementary kids at a monastery school, providing monthly medical checkups in slum areas, and working as head of PR for a non-profit library. My experience working in the slums inspired my current path. I decided to pursue Chemistry because I wanted to develop affordable and effective therapeutics for common diseases in developing countries like Myanmar. Receiving a generous full-tuition scholarship to study at Berea College in 2012 was a turning point in my life. From there, I graduated with a B.A. in Chemistry and Computer Science in 2016. Then, I joined Dr. Fourches’ lab at North Carolina State University and completed a Ph.D. in Chemistry (Cheminformatics) in 2020.

As part of my thesis, I developed algorithms and tools to design large libraries of macrolides/macrocycles. Additionally, I developed CryptoChem, an encryption technology to securely store and transfer information using chemicals. I also designed and developed MD-QSAR technology which combined machine learning, 3D docking, and molecular dynamics to calculate complex target endpoints. As part of my internship at Collaborations Pharmaceuticals during my Ph.D., I built MacrolactoneDB, a comprehensive database of curated macrolactones with bioactivities across targets, conducted ML studies, and developed a pipeline to estimate the applicability and reliability domains for the in-house models. After completing my Ph.D. I worked for two years as a Cheminformatics Postdoc/ Scientist I in the ADMET Predictor team at Simulations Plus, Inc.

## What is your current research focus, and what are your plans for the future?

PPKZ: My current research is quite Terray specific. Among many things, I design diverse and focused screening libraries which contain up to hundreds of millions of molecules that are to be synthesized on chips. I also develop rapid, reaction-based enumeration methods for enumerating virtual libraries, analyze large chemical data, and extract SAR (structure–activity relationships) and chemical insights for interactive drug design and optimization. Furthermore, I work with building block vendors to characterize their catalogs and help with purchases to expand the Terray chemical space.

My plans for the long term involve developing state-of-the-art bio-pharmacokinetic simulations and automated drug discovery pipelines. This will allow us to identify lead therapeutics efficiently and accurately for any diseases or biological targets of interest. It is more of a vision because it will require advanced cheminformatics techniques, medicinal chemistry, and biology expertise, as well as substantial amounts of genetic, biological, and chemical data. Taking it a step further, I envision working on providing precision & personalized medicine to cure diseases with minimal adverse reactions and side effects. e.g., predicting properties such as ADMET (Absorption, Distribution, Metabolism, Excretion, and Toxicity) of drugs for individuals by taking account of their DNA gene variants.

## Which obstacles did you encounter during your career, and what experiences have helped you get to where you are today?

PPKZ: Personally, self-doubt was one of the main *internal* obstacles that I encountered in my career. When I talked to colleagues during my Ph.D., I found that it is something many of us shared, e.g., not thinking your project is worth publishing, doubting your skills and struggling to accept your success when they call your name in award ceremonies. I came to know that as imposter syndrome.

Maybe it stems from external factors such as cultural upbringing, systematic oppression, and gender biases. I grew up in a conservative culture under a military dictatorship where people believe men are born with greater spiritual power (or “hpone”) than women. Even in daily chores, we do not mix men’s and women’s garments because it is believed it could lower men’s hpone. If you grow up experiencing such treatments everywhere, your self-image will be heavily influenced. If girls are being told they are less than men from the moment they are born, they might just believe it themselves. You can read more about it in a relevant blog post [2] that I wrote on my personal website [3].

In my case, recognizing self-doubt and consciously deciding to become confident helped me overcome it. It is important to encourage and advocate for yourself because society may not do that for you. I am ambitious and pertinacious, so if self-doubt or fear starts to linger in my head, I kick them to the curb. And I benefit greatly from that because I have won prestigious scholarships and accolades in competitions, I didn’t think I deserve. I am also fortunate to have met many kind and supportive mentors, which play a key role in overcoming imposter syndrome. It is also immensely helpful to have a great partner, and I thank my husband for his dedicated support, and empowerment throughout the years. I am also grateful to my family for their unconditional love and support, which shaped me to be who I am today.

## What advice would you give to your younger self?

PPKZ: A lot, and I am still in the early stages of my career! I would advise my younger self to embrace the mistakes and be less critical of myself. I used to strive for perfectionism, and it held me back in some ways. Now, I give my best at whatever I set my mind to, instead of spending too much time perfecting or worrying about it. Making mistakes is crucial in the learning and growing process, and it is only human to err. In general, it is important to be kind to ourselves and reduce unnecessary stress or worries.

I would also tell my younger self to be bolder, and more confident, which is easier said than done. I came from a culture that is more reserved, and soft-spoken, and I regard some aspects of it fondly. However, when it comes to career, it is important to be fearless and assertive, especially about the projects you want, boundaries, and career negotiations.

## What is a current challenge you are facing that should not be a challenge in the near future?

PPKZ: A current challenge I face in the Cheminformatics field is the scarcity of high-quality chemical/biological data. I agree with a former interviewee, Dr. Emma Schymanski [1], that we spend a large amount of time extracting and curating chemical/biological data from literature and public databases. In my role at Simulations Plus Inc., I worked on producing high-quality data, modeling ADMET properties and toxicity endpoints, and predicting metabolism. As you can imagine, a large amount of effort is invested in the data mining and curation pipeline. You can check out a couple of my presentations here: “Untold Stories of Data Curation—MDCK Data Curation and Modeling” [4] and “How we leverage automation for data mining, preprocessing and curation” [5].

It is not incredibly difficult to build a good machine learning model given a proper amount of well-curated chemical data, and the pipeline for building and validating models can be automated. What is rather difficult is extracting and curating the underlying data which is essential for model-building. If a model is not performing well, quite often, the issue is with the underlying data or descriptors, which may contain irrelevant information, mistakes in the structures, endpoints, cell lines, substrates, or a lot of noise. The point is, you cannot build a good model if a good chunk of your data is junk, and the model can only be as good as the data you use to train it. So, it is important to do a thorough analysis to make sure you have high-quality, extensive data.

My current work at Terray Therapeutics helps with overcoming the data scarcity challenge in the field because we are generating extremely large, precise chemical datasets for biological targets of interest at an unparalleled scale using microarray technology. High-diversity compound libraries containing up to millions of small molecules are synthesized, screened, and measured in minutes, so it is extremely fast. We have a closed-loop iterative system because we create a ton of data, test them in the lab, model them in-silico, and validate them again experimentally within weeks or days, which is very different from the traditional drug discovery workflow.

I am excited about exploring and better understanding the vast chemical space because we can eventually map it out and unlock important pharmacokinetic insights to deliver treatments to otherwise inaccessible targets in an accelerated timeline. Also, which cheminformatics scientist wouldn’t dream of applying newly discovered SAR insights in the next cycle of drug design, building models, making predictions, and having them evaluated in real life?

## What do you think the cheminformatics community could do to increase diversity and inclusion?

PPKZ: Open science and access to knowledge are crucial for inclusion because it tears down financial barriers. Scholarships or small funds to cover career or exam-related expenses are very helpful, especially for minorities and those from developing countries. It was life-changing in my case. I know a lot of international fellows from low economic backgrounds who were given an opportunity to pursue high-quality education through similar scholarships. It is inspiring to see them now thriving successfully in their respective fields and contributing back to their communities. It is also a good idea to reach out to women and/or minorities to give talks, collaborate on papers, participate in peer reviews, and do activities as part of the community.

Early in 2022, I participated in the Women in Pharmaceutical Science panel from Simulations Plus Inc. and it was a wonderful and empowering experience. We talked about the challenges we face in the workplace including topics on implicit biases, imposter syndrome, life/work balance, and mentorship programs. Holding such panels brings awareness and togetherness to the community through sharing of firsthand experiences and suggestions that are practical and useful.

## Can you share one recent source of inspiration for your scientific work?

PPKZ: The outbreak of Covid-19 global pandemic not only signifies an urgent need to accelerate the drug discovery pipeline, but also highlights the instrumental role of computational tools in the unprecedented speed for vaccine development. It is inspiring to see the science community from all around the world come together to beat this pandemic and develop effective vaccines in an incredibly short timeline. It deeply inspires me, on a personal level, because I came into this field wanting to develop safe, inexpensive, and potent therapeutics in an expedited timeline for common diseases such as malaria, tuberculosis, dengue fever, and hepatitis in developing countries. And I see this miracle happen in the early years of my career. I am excited and thankful to be in this field, and I have a lot of passion for the work that I do. My current work aligns well with my long-term vision, and it gives me an incredible opportunity to learn, explore, better understand the vast chemical space, and discover pharmacokinetic insights crucial in the drug development. There is so much we still don’t know. The core of cheminformatics is founded on the notion that similar molecules have similar properties (SAR), except for activity cliffs, and I am excited to be working on its frontier.

## Photos


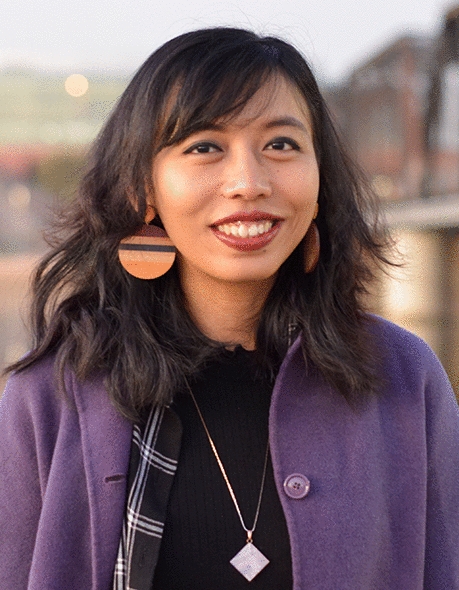


## Data Availability

Not applicable.
